# MIS‐C Followed by Systemic Juvenile Idiopathic Arthritis: A Diagnostic Challenge in a Child

**DOI:** 10.1155/crpe/5261060

**Published:** 2026-11-09

**Authors:** Elda Skenderi, Gjeorgjina Kuli-Lito, Alberta Shkembi

**Affiliations:** ^1^ University Hospital Center “Mother Teresa” Tirana Albania

**Keywords:** diagnostic challenge, multisystem inflammatory syndrome in children (MIS-C), recurrent fever, SARS-CoV-2, systemic-onset juvenile idiopathic arthritis (sJIA)

## Abstract

**Background:**

Multisystem inflammatory syndrome in children (MIS‐C) is a severe postinfectious inflammatory condition associated with SARS‐CoV‐2 infection and characterized by immune dysregulation with hyperinflammation and multisystem involvement. Its clinical manifestations may overlap with other pediatric inflammatory disorders, including systemic‐onset juvenile idiopathic arthritis (sJIA), creating diagnostic challenges.

**Case Presentation:**

We report the case of a 9‐year‐old child who presented with a 7‐day history of high fever, fatigue, myalgia, arthralgia, anorexia, gastrointestinal symptoms, and a transient maculopapular rash. Laboratory investigations revealed marked systemic inflammation, including leukocytosis, neutrophilia, elevated C‐reactive protein, ferritin, and fibrinogen levels. The patient also had gastrointestinal, mucocutaneous, and respiratory involvement, with positive anti‐SARS‐CoV‐2 IgG antibodies. Based on the clinical and laboratory findings, MIS‐C was diagnosed and treated with intravenous immunoglobulin and systemic corticosteroids, resulting in complete clinical recovery and normalization of inflammatory markers. Approximately 2 weeks after discharge, the child was readmitted with recurrent high fever, fatigue, myalgia, arthralgia, and anorexia. Inflammatory markers were again markedly elevated, with a substantial increase in ferritin levels. Musculoskeletal ultrasonography demonstrated synovial inflammation and joint effusion involving the knee and sacroiliac joints. In light of the emergence of objective synovitis and persistent systemic inflammation, the diagnosis was revised to sJIA. Treatment with nonsteroidal anti‐inflammatory drugs, systemic glucocorticoids, and methotrexate resulted in clinical improvement and subsequent remission.

**Conclusion:**

The recurrence of fever and systemic inflammation after apparent clinical and laboratory resolution of MIS‐C should prompt careful reassessment of the diagnosis and consideration of other inflammatory disorders. The subsequent emergence of objective arthritis or synovitis may be an important clue to underlying sJIA.

## 1. Introduction

Soon after the global outbreak of COVID‐19, several countries reported an increasing number of children developing a novel multisystem inflammatory condition occurring several weeks after SARS‐CoV‐2 infection. This condition, subsequently termed multisystem inflammatory syndrome in children (MIS‐C), is characterized by persistent fever, marked systemic inflammation, and involvement of multiple organ systems. Its clinical manifestations may include gastrointestinal symptoms such as diarrhea, vomiting, and abdominal pain; mucocutaneous findings including rash, conjunctival injection, and edema; and respiratory, cardiovascular, or neurological involvement. Severe cases may be complicated by shock and frequently require intensive care [1, 2].

Systemic‐onset juvenile idiopathic arthritis (sJIA) is a subtype of JIA, a heterogeneous group of chronic inflammatory arthritides affecting children. Unlike most JIA subtypes, which are primarily considered autoimmune disorders, sJIA is classified as an autoinflammatory disease characterized predominantly by dysregulation of the innate immune system [3]. The clinical presentation of sJIA may include quotidian or intermittent high‐grade fever, fatigue, anorexia, weight loss, myalgia, arthralgia, evanescent rash, lymphadenopathy, hepatosplenomegaly, serositis, and markedly elevated inflammatory markers. There is no single diagnostic test for sJIA, and laboratory abnormalities are nonspecific. Systemic manifestations may precede the development of clinically apparent arthritis, which can make the diagnosis particularly challenging during the early phase of the disease [4].

MIS‐C and sJIA share several clinical and laboratory features, including persistent fever, rash, gastrointestinal manifestations, musculoskeletal symptoms, and marked systemic inflammation. This overlap can complicate the differential diagnosis, particularly when arthritis is absent at initial presentation [5]. In this case report, we describe a child initially diagnosed with MIS‐C who experienced complete clinical and laboratory recovery following treatment but subsequently developed recurrent systemic inflammation and objective synovitis, leading to a revised diagnosis of sJIA. The case highlights the importance of continued clinical reassessment when systemic inflammatory manifestations recur after apparent resolution of MIS‐C.

## 2. Case Report

A 9‐year‐old school‐aged child was admitted to the General Pediatric Ward in December 2024 with a 7‐day history of high‐grade fever, fatigue, myalgia, arthralgia, and loss of appetite. During the preceding 2 days, a maculopapular rash had developed on the trunk and extremities and became more prominent during fever peaks. The child had initially received oral antibiotics at a local clinic; however, the fever persisted despite treatment and was poorly responsive to antipyretics. The persistence of the high‐grade fever and the development of a rash prompted admission to a tertiary‐care hospital.

On admission, the child appeared moderately ill. The main symptoms were fatigue, generalized muscle and joint pain, anorexia, nausea, and abdominal pain. The fever reached 39.5°C, occurring two to three times daily despite treatment with acetaminophen and ibuprofen. There was no neck stiffness or other neurological abnormality. Mild scleral injection and pharyngeal hyperemia without exudates were observed. There was no cervical lymphadenopathy or peripheral edema. A mildly pruritic, blanching, transient maculopapular rash was present predominantly on the trunk, and to a lesser extent, on the face and extremities (Figure 1). The respiratory rate was approximately 50 breaths/min, and fine bilateral rales were heard on auscultation; oxygen saturation was preserved (98%) in room air. The heart rate was 120 beats/min. The abdomen was soft, with normal bowel sounds, and no hepatosplenomegaly was detected on palpation.

**FIGURE 1:**
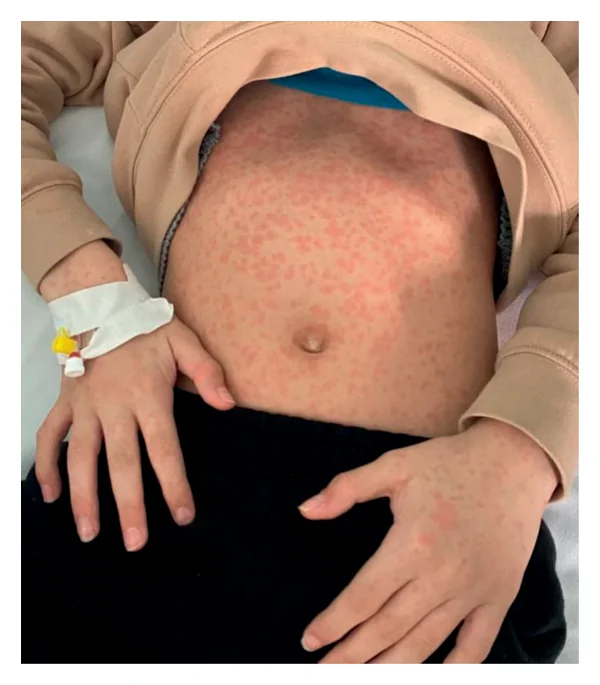
Maculopapular rash spread over trunk, face, and extremities.

The child had previously been healthy and was the youngest member of the family. The parents and two older siblings were healthy. Routine vaccinations were up to date according to the national immunization schedule, which at that time did not include SARS‐CoV‐2 vaccination. There was no history of domestic animal exposure, and the family reported consumption of food from safe sources.

### 2.1. Investigations and Initial Diagnosis

Initial investigations included a complete blood count, biochemical profile, microbiological cultures, serological testing, a tuberculin skin test, and thoracic and abdominal imaging. Microbiological and serological investigations included throat, blood, urine, and stool cultures, as well as testing for Leishmania, Salmonella, Brucella, Rickettsia, HIV, Epstein–Barr virus (EBV), cytomegalovirus (CMV), and SARS‐CoV‐2.

Laboratory investigations demonstrated marked leukocytosis and neutrophilia, accompanied by elevated C‐reactive protein and ferritin levels, and a less prominent increase of NT‐pro‐BNP, erythrocyte sedimentation rate (ESR), and fibrinogen levels, whereas liver and renal function were preserved (Table 1). Chest radiography revealed peribronchial inflammatory changes. Abdominal ultrasonography demonstrated mildly enlarged mesenteric lymph nodes, while the liver and spleen were normal in size and appearance. Echocardiography demonstrated normal cardiac structure and function.

**TABLE 1:** Laboratory results on first admission.

Parameter	Patient value	Reference range
WBC	31,000 cells/mm^3^	4000–12,000 cells/mm^3^
Neutrophils	23,500 cells/mm^3^	920–6000 cells/mm^3^
Lymphocytes	4000 cells/mm^3^	1840–9120 cells/mm^3^
RBC	3,560,000 cells/mm^3^	4,000,000–5,000,000 cells/mm^3^
Haemoglobin	10.2 g/dL	10.5–12.7 g/dL
Haematocrit	28.5%	33%–43%
PLT	321,000 cells/mm^3^	150,000–400,000 cells/mm^3^
Erythrocyte sedimentation rate	31 mm/h	< 15 mm/h
Aspartate aminotransferase	38 U/L	20–45 U/L
Alanine aminotransferase	19 U/L	15–25 U/L
Blood urea nitrogen	10.2 mg/dL	10.9–36 mg/dL
Creatinine	0.38 mg/dL	0.38–0.54 mg/dL
Lactate dehydrogenase	319 U/L	170–350 U/L
C reactive protein	10.23 mg/dL	< 0.5 mg/dL
Fibrinogen	461 mg/dL	130–420 mg/dL
Ferritin	970 ng/mL	15–90 ng/mL
Albumin	4.1 g/dL	3.8–5.4 g/dL
NT‐pro‐BNP	510 pg/mL	< 125 pg/mL

Empirical treatment with a third‐generation cephalosporin was initiated. However, the fever persisted, and there was no significant clinical improvement. All cultures remained negative for bacterial growth. Serological tests for Leishmania, Salmonella, Brucella, Rickettsia, HIV, EBV, and CMV were negative, as was the tuberculin skin test.

Given the persistent fever, marked systemic inflammation, mucocutaneous, gastrointestinal, and respiratory manifestations, and the absence of evidence of an infectious cause, the differential diagnosis shifted toward a systemic inflammatory syndrome. At this stage, sJIA was not considered the most likely diagnosis because objective joint involvement was absent. Kawasaki disease was also considered because of the presence of fever, rash, and conjunctival injection; however the overall clinical and laboratory profile did not fulfill the diagnostic criteria for complete or incomplete Kawasaki disease. Macrophage activation syndrome was also considered because of the high ferritin concentration and systemic inflammatory response. However the available laboratory findings did not support MAS, as the patient had preserved platelet counts, elevated rather than decreased fibrinogen, and no evidence of significant hepatic dysfunction [6].

Serological testing for SARS‐CoV‐2 revealed positive anti‐SARS‐CoV‐2 IgG antibodies (130.7 RU/mL). Based on the clinical presentation, laboratory evidence of systemic inflammation, multisystem involvement, and positive SARS‐CoV‐2 serology in an unvaccinated child, MIS‐C was strongly considered. The patient fulfilled the clinical and laboratory criteria of the 2023 Council of State and Territorial Epidemiologists (CSTE) and Centers for Disease Control and Prevention (CDC) surveillance case definition for MIS‐C. He had documented a fever ≥ 38°C, illness requiring hospitalization, marked systemic inflammation with a C‐reactive protein level of 10.23 mg/dL, and new‐onset mucocutaneous and gastrointestinal involvement, manifested by a transient maculopapular rash, conjunctival injection, nausea, and abdominal pain. SARS‐CoV‐2 was positive for anti‐SARS‐CoV‐2 IgG (130.7 RU/mL) [2]. The diagnosis of MIS‐C was established after exclusion of alternative infectious and inflammatory diagnoses. Although NT‐pro‐BNP was elevated, echocardiography was normal, and the elevated NT‐pro‐BNP was considered supportive rather than a formal cardiac criterion of the 2023 CSTE/CDC surveillance definition.

### 2.2. Treatment and Clinical Response

The patient was treated with intravenous immunoglobulin (IVIG) at a dose of 2 g/kg administered as a continuous infusion over 36 h and intravenous prednisolone at 1 mg/kg daily for 5 days. Following treatment, the fever resolved, the rash disappeared, inflammatory markers normalized, and the child’s general condition returned to baseline. The patient was subsequently discharged from the hospital.

### 2.3. Recurrence of Symptoms and Revised Diagnosis

Approximately 2 weeks after discharge, the child was readmitted because of recurrent persistent high‐grade fever, fatigue, myalgia, arthralgia, difficulty climbing stairs, nausea, and loss of appetite. On examination, the patient appeared ill and weak, with tenderness on the joints and muscles on palpation.

Repeat laboratory investigations demonstrated recurrent and marked systemic inflammation, including leukocytosis, neutrophilia, elevated ESR, C‐reactive protein, fibrinogen, and ferritin levels (Table 2). Rheumatoid factor (RF) and antinuclear antibodies (ANA) were negative.

**TABLE 2:** Laboratory results on second admission.

Parameter	Patient value	Reference range
WBC	31,200 cells/mm^3^	4000–12,000 cells/mm^3^
Neutrophils	25,000 cells/mm^3^	920–6000 cells/mm^3^
RBC	3,600,000 cells/mm^3^	4,000,000–5,000,000 cells/mm^3^
Haemoglobin	11.0 g/dL	10.5–12.7 g/dL
PLT	325,000 cells/mm^3^	150,000–400,000 cells/mm^3^
ERS	61 mm/h	< 15 mm/h
CRP	13.2 mg/dL	< 0.5 mg/dL
Fibrinogen	623 mg/dL	130–420 mg/dL
Ferritin	2740.54 ng/mL	15–90 ng/mL
Rheumatoid factor	< 20 IU/mL	< 30 IU/mL
ANA	Negative	< 160
NT‐pro‐BNP	115 pg/mL	< 125 pg/mL
Aspartate aminotransferase	41 U/L	20–45 U/L
Alanine aminotransferase	17 U/L	15–25 U/L

Musculoskeletal ultrasonography of the knee and sacroiliac joints demonstrated synovial inflammation with 4–5 mm joint effusion. Abdominal ultrasonography showed mild enlargement of the liver and spleen.

The recurrence of persistent high‐grade fever and systemic inflammation, together with myalgia, arthralgia, and newly documented objective synovitis, prompted reconsideration of the initial diagnosis. The clinical and laboratory findings were considered consistent with sJIA, and the diagnosis was revised accordingly. Given the extensive elevation of ferritin (2740.54 ng/mL), MAS was reconsidered in the second admission; however, it was still not supported by laboratory findings, preserved platelet counts, elevated rather than decreased fibrinogen, and a normal level of hepatic enzymes (ALT, AST).

### 2.4. Treatment and Follow‐Up

Treatment with nonsteroidal anti‐inflammatory drugs (ibuprofen 40 mg/kg/day), high‐dose systemic glucocorticoids (methylprednisolone 30 mg/kg/day for 3 days), and disease‐modifying antirheumatic drugs (methotrexate 15 mg/m^2^ weekly) was initiated. Following initial treatment for sJIA, the patient’s fever resolved by the fifth day, accompanied by progressive improvement in fatigue, myalgia, arthralgia, and mobility. Serial laboratory testing demonstrated a gradual decrease in inflammatory markers and ferritin levels. Prednisone was progressively tapered over 4 weeks, while methotrexate was continued as maintenance therapy.

During subsequent follow‐up, the patient remained clinically well, without recurrence of high‐grade fever or significant joint symptoms. At the latest follow‐up, approximately 16 months after the second admission, the patient had remained in clinical remission under rheumatologic follow‐up (Table 3). No major adverse events related to treatment were documented. The favorable clinical course, including sustained clinical remission during follow‐up, supports the effectiveness of the treatment strategy used in this individual patient. However, this does not establish methotrexate‐based therapy as the preferred contemporary first‐line approach for sJIA, current international recommendations favor IL‐1 or IL‐6 inhibition as an important treatment option [7].

**TABLE 3:** Timeline of the case.

Time point	Clinical findings	Investigations	Diagnosis	Treatment	Outcome
Day‐7 to 0	High‐grade fever, fatigue, myalgia, anorexia, transient rash	Elevated CRP, ferritin, fibrinogen, NT‐pro‐BNP, positive SARS‐CoV‐2 IgG	MIS‐C considered/diagnosed	IVIG + prednisolone	Clinical and laboratory improvement
Approximately 2 weeks later	Recurrent fever, fatigue, myalgia, arthralgia, difficulty climbing stairs	CRP, ESR, and ferritin, markedly increased; ultrasound showed synovial inflammation and effusion	Diagnosis reconsidered; sJIA diagnosed	Ibuprofen + IVmethylprednisolone + methoterexate	Fever resolved; inflammatory markers decreased
Follow‐up	Clinical remission	—	sJIA in remission	Methotrexate maintenance	Sustained remission

## 3. Discussion

MIS‐C emerged shortly after the COVID‐19 outbreak as a severe inflammatory condition in children characterized by persistent fever, systemic inflammation, and multisystem involvement. It is considered a postinfectious inflammatory condition in which a SARS‐CoV‐2 may trigger dysregulated immune responses and widespread inflammation. Several mechanisms have been proposed, including excessive T‐cell activation, cytokine production, and autoreactive immune responses. The SARS‐CoV‐2 spike protein has also been proposed to possess superantigen‐like properties that may contribute to immune activation. Studies have additionally demonstrated autoantibodies targeting antigens expressed in endothelial and gastrointestinal tissues, as well as antigens associated with rheumatologic conditions. Genetic susceptibility may also contribute to the development of MIS‐C, with specific HLA Class I alleles having been implicated as potential risk factors [8–10].

There is no single diagnostic test for MIS‐C. Diagnosis relies on the combination of persistent fever, systemic inflammation, multisystem involvement, evidence of recent SARS‐CoV‐2 infection, and exclusion of alternative infectious and inflammatory diagnoses. In the present case, there was no history of a recent symptomatic SARS‐CoV‐2 infection; however, anti‐SARS‐CoV‐2 IgG antibodies were detected, suggesting previous exposure. The absence of vaccination further supported the interpretation of the positive serology as evidence of previous infection.

At the first admission, the patient presented with high‐grade fever, marked systemic inflammation, and new‐onset mucocutaneous and gastrointestinal manifestations, together with positive SARS‐CoV‐2 serology. The predominant clinical manifestations of MIS‐C involve the gastrointestinal, cardiovascular, hematologic, and mucocutaneous systems. Involvement of the respiratory tract, central nervous system, and renal system occurs with less frequency. Parenchymal pulmonary involvement is comparatively less common in MIS‐C and can confound the initial clinical presentation by mimicking primary acute respiratory tract infections. The extensive infectious workup was negative, making bacterial sepsis and several other infectious conditions less likely. The respiratory findings were mild, with preserved oxygenation and no need for respiratory support. Nevertheless, the increased respiratory rate, bilateral fine rales, and radiographic prominence of the bronchial markings represented an additional organ‐system finding within the initial multisystem inflammatory presentation, while the absence of hypoxemia or respiratory distress made significant primary pulmonary disease less likely. The elevated NT‐pro‐BNP provided laboratory evidence of cardiac involvement despite normal cardiac structure and function on echocardiography. Kawasaki disease, toxic shock syndrome, and macrophage activation syndrome were also considered but were not supported by the overall clinical presentation and laboratory parameters. These findings led to the initial diagnosis of MIS‐C.

The subsequent clinical course, however, raised an important diagnostic question. Approximately 2 weeks after apparent clinical and laboratory recovery following treatment for MIS‐C, the patient developed recurrent high‐grade fever, systemic symptoms, and markedly increased inflammatory markers. At this second presentation, musculoskeletal manifestations had become more prominent, and ultrasonography demonstrated synovial inflammation and joint effusion. This emergence of objective joint involvement was a critical finding that prompted reconsideration of the diagnosis.

The diagnosis of sJIA is similarly based on clinical assessment and exclusion of alternative diagnoses, as there is no specific diagnostic test. Persistent high‐grade fever is one of its most characteristic presenting manifestations and may be accompanied by rash, myalgia, arthralgia, lymphadenopathy, hepatosplenomegaly, serositis, and marked elevation of inflammatory markers. Systemic manifestations may precede the development of objective arthritis. Arthralgia may occur before clinically evident arthritis, while arthritis itself may develop later in the disease course [3, 4]. In the present case, the absence of objective arthritis during the first admission made sJIA difficult to establish at that stage. The initial presentation nevertheless shared several features with sJIA, including prolonged high‐grade fever, transient rash, musculoskeletal symptoms, and pronounced systemic inflammation. During the second admission, the appearance of synovitis and joint effusion, together with recurrent fever and worsening inflammatory markers, provided additional evidence supporting sJIA.

The overlap between MIS‐C and sJIA may be partly explained by their shared inflammatory pathways. Systemic JIA is considered an autoinflammatory disorder in which dysregulation of the innate immune system leads to excessive production of proinflammatory cytokines. Interleukin‐1 and interleukin‐6 have important roles in the pathogenesis of sJIA and contribute to fever, anorexia, pain, acute‐phase responses, and other systemic manifestations [11, 12]. Similarly, dysregulated cytokine responses, including IL‐6 activity, have been described in MIS‐C. Thus, the clinical and laboratory similarities between these conditions may make their distinction particularly difficult when arthritis is not yet objectively apparent. Hyperferritinemia is a common feature of both disorders and reflects the intensity of systemic inflammation rather than being disease‐specific. In the present case, ferritin was markedly elevated during both episodes, reaching 970 ng/mL during the initial presentation and 2740.54 ng/mL during recurrence. The substantially higher ferritin concentration during the second episode, together with the development of objective synovitis, contributed to reconsideration of the initial diagnosis. The normalization of NT‐pro‐BNP during the second admission also provided a temporal distinction between the two episodes. During the initial illness, NT‐pro‐BNP was elevated despite normal echocardiographic findings, whereas it had decreased to within normal range at recurrence. In contrast, inflammatory and ferritin markers were markedly increased during the second episode, coinciding with the emergence of articular manifestations. These observations support the concept that biomarkers should be interpreted in the context of the clinical phenotype and disease trajectory rather than in isolation.

The diagnostic overlap between MIS‐C and sJIA has been increasingly recognized since the emergence of SARS‐CoV‐2. Published reports have described children with prolonged or recurrent fever, rash, gastrointestinal manifestations, marked hyperinflammation, and hyperferritinemia in whom the distinction between MIS‐C and sJIA was initially difficult. In particular, recurrent or persistent fever despite apparent resolution of the initial inflammatory episode, together with the subsequent emergence of arthritis, has been suggested as an important clue toward sJIA [5]. Previous case reports have also described sJIA initially diagnosed as MIS‐C because of a history of SARS‐CoV‐2 exposure or positive serology, systemic inflammation, and overlapping clinical manifestations. These observations emphasize that positive SARS‐CoV‐2 antibodies alone cannot establish MIS‐C when the subsequent clinical course is inconsistent with the expected evolution of the syndrome [13]. The present case differs in that the patient initially demonstrated a clinical course compatible with MIS‐C, including complete clinical and laboratory improvement after IVIG and corticosteroid therapy. The subsequent episode occurred after an interval of apparent remission.

An important feature of this case is the transition from an initial MIS‐C phenotype to a subsequent presentation compatible with sJIA. The temporal association and the emergence of objective synovitis after resolution of the initial inflammatory syndrome highlight the need for continued clinical surveillance and diagnostic reassessment when systemic inflammatory symptoms recur. This case emphasizes that a previous diagnosis of MIS‐C should not prevent clinicians from reconsidering alternative inflammatory diagnoses when the clinical course becomes atypical. In particular, recurrence of fever and marked inflammation after apparent resolution of MIS‐C, especially when accompanied by persistent musculoskeletal symptoms and objective synovitis, should prompt evaluation for sJIA and other systemic inflammatory diseases.

## 4. Conclusion

The recurrence of high‐grade fever and systemic inflammation after apparent clinical and laboratory resolution of MIS‐C should prompt careful diagnosis reassessment. The emergence of objective arthritis or synovitis may provide an important clue to an underlying systemic inflammatory disorder such as sJIA. This case highlights the importance of longitudinal clinical follow‐up and maintaining a broad differential diagnosis when the subsequent clinical course is inconsistent with the expected evolution of MIS‐C.

## 5. Strengths and Limitations

### 5.1. Strengths

• The case illustrates a clinically challenging overlap between MIS‐C and sJIA, two inflammatory conditions with several shared systemic manifestations.
• The longitudinal clinical course allowed reassessment of the initial diagnosis when new objective musculoskeletal findings emerged.
• Musculoskeletal ultrasonography provided objective evidence of synovial inflammation and joint effusion, supporting the subsequent diagnosis of sJIA.
• The patient had clinical improvement following treatment for sJIA and remained in clinical remission during follow‐up.

### 5.2. Limitations

• As a single case report, this observation cannot establish a causal relationship between SARS‐CoV‐2 exposure, MIS‐C, and the subsequent development or clinical expression of sJIA.
• The absence of objective arthritis during the first admission makes it difficult to determine retrospectively whether sJIA was already developing at the time of the initial MIS‐C presentation.
• The diagnosis of MIS‐C and the subsequent diagnosis of sJIA were based on clinical, laboratory, and imaging findings rather than disease‐specific biomarkers.
• The case therefore highlights a diagnostic association and clinical evolution rather than proving that MIS‐C preceded or triggered sJIA.

## Funding

No funding was received for this manuscript.

## Ethics Statement

The authors have nothing to report.

## Consent

Written informed consent was obtained from the patient’s parent for publication of this case report and the accompanying image, providing anonymity.

## Conflicts of Interest

The authors declare no conflicts of interest.

## Data Availability

The data that support the findings of this study are available from the corresponding author upon reasonable request.
